# What do stroke survivors’ value about participating in research and what are the most important research problems related to stroke or transient ischemic attack (TIA)? A survey

**DOI:** 10.1186/s12874-021-01390-y

**Published:** 2021-10-10

**Authors:** Ishanka Weerasekara, Jasmine Baye, Meredith Burke, Gary Crowfoot, Gillian Mason, Rachael Peak, Dawn Simpson, Frederick Rohan Walker, Michael Nilsson, Michael Pollack, Coralie English

**Affiliations:** 1 School of Health Sciences, College of Health, Medicine and Wellbeing & Priority Research Centre for Stroke and Brain Injury, The University of Newcastle, Callaghan, Australia; 2 Department of Physiotherapy, Faculty of Allied Health Sciences, University of Peradeniya, Peradeniya, Sri Lanka; 3 Hunter Medical Research Institute, New Lambton Heights, Australia; 4 Newcastle, Australia; 5 School of Nursing and Midwifery and Priority Research Centre for Stroke and Brain Injury, The University of Newcastle, Callaghan, Australia; 6 Centre for Research Excellence in Stroke Recovery and Rehabilitation, Florey Institute of Neuroscience and Hunter Medical Research Institute, Parkville, Australia; 7 Centre for Rehab Innovations, School of Medicine and Public Health, College of Health Medicine and Wellbeing, The University of Newcastle, Callaghan, Australia; 8 Rehabilitation Medicine, John Hunter Hospital, New Lambton Heights, Australia; 9 School of Medicine and Public Health, The University of Newcastle, Callaghan, Australia

## Abstract

**Background:**

Recruitment to stroke clinical trials is challenging, but consumer registers can facilitate participation. Researchers need to understand the key factors that facilitate trial involvement and improve consumer partnerships to identify what research topics important to stroke and transient ischemic attack (TIA) survivors and their carers. We aimed to examine i) the experience of being involved in a stroke research register, and ii) the priorities for stroke research from the perspective of stroke survivors.

**Methods:**

Online and paper-based surveys were sent directly to members of a stroke register and disseminated online. Multiple choice questions were reported as counts and percentages and open-ended questions were thematically analysed using Braun and Clarke’s 6-stage process.

**Results:**

Of 445 survey respondents, 154 (38%) were a member of the Stroke Research Register. The most frequently reported reason for research participation was to help others in the future. Respondents reported they were less likely to take part in research if the research question was not relevant to them, if transport was an issue, or because they lacked time. The most important research problems reported were targeting specific impairments including recovery of movement, fatigue, and aphasia, improvement of mental health services, and increased support for carers.

**Conclusions:**

Recruitment to trials may be improved by research registers if an inclusive research culture is fostered, in which consumers feel valued as members of a community, have direct and timely access to research findings and the opportunity to be meaningfully involved in research around the problems that consumers find most important.

**Supplementary Information:**

The online version contains supplementary material available at 10.1186/s12874-021-01390-y.

## Introduction

Stroke is a leading global cause of disability [1]. Despite advances in stroke care improving health outcomes for stroke survivors, new therapies and preventative measures remain a major research priority [2]. Trial recruitment is critical to completion of robust research studies. Failure to meet recruitment targets on time (or at all) is a major contributor to research inefficiency and waste [3] with approximately half of all studies failing to recruit their target sample size [4]. Reasons for inefficient trial recruitment include complexities of ethics and regulatory approvals, particularly for multicenter trials [3], and a lack of flexibility in informed consent procedures. Genuine partnerships between consumers (The term ‘consumer’ was used as we believe that it accommodates shared decision-making and the commodification of healthcare) and researchers in all phases of research may improve recruitment rates. A review of 26 studies conducted between 2000 and 2017 found that consumer involvement in trials increased the likelihood of meeting recruitment targets on time [5]. This effect was amplified when consumers with relevant lived experience were involved with the design and implementation of a trial [5].

Several studies have sought to investigate barriers and solutions to clinical trial recruitment from the perspectives of researchers’, clinicians’ [6, 7], or by identifying reasons for refusal from trial screening logs [8]. Clinical research to deliver answers to relevant questions of interest and ultimately improve the health and well-being of people. To do this, researchers need to understand the key reasons that people participate in studies, how involvement in trials can be facilitated, and how researchers can partner with consumers to ask the right questions, in the right way, and at the right time.

In 2016, we set up the Stroke Research Register, Hunter (‘Register’) with the aim of improving consumer engagement in research and supporting timely recruitment of stroke survivors to clinical trials. The Register is a database of individual members who have experienced a stroke or transient ischemic attack (TIA) that have provided consent to be contacted about research projects. Since its formation, the Register has assisted in recruiting approximately 230 individual participants to 13 different clinical trials. The Register has also facilitated consumers to be involved as research partners in 8 different research teams.

The aim of this study was to evaluate the consumer experience of being a Register member and of participating in research. A further aim was to explore priorities for research from the perspective of people with lived experience of stroke.

The research questions were:

1. What do stroke survivors’ value about being a member of the Stroke Research Register?
2. What are stroke survivors’ top reasons for taking part (or not taking part) in research studies?
3. What are stroke survivors’ preferred communication methods with researchers?
4. What are stroke survivors’ most important research problems related to stroke or transient ischemic attack (TIA)?

## Method

A survey was conducted between May 21st, 2019 to July 29th, 2019. The survey was sent to all members (*n* = 513) of the Register via email or hard copy. The survey was promoted on social media with a direct link to an online version. The responses from the online version were stored on *Qualtrics* (Provo, Utah, USA) with the paper-based surveys returned by mail or email. The Hunter region, or the Hunter, is a region in the state of New South Wales (NSW) Australia and this is one of the largest river valleys on the NSW coast.

The survey was designed to be aphasia friendly by a research team including researchers and people with lived experience of stroke. This was achieved through the addition of emoticons and icons next to answers along with bolding of key words in the questions and answers. The questionnaire layout used lots of white space around the questions with simple borders and bigger fonts for key words and headings. Questions and answers were structured to use short simple language to be easily understood. Open-ended questions were given a large box with no word limit. Open-ended questions were selected for questions about why a respondent choose to participate (or not) in research and about what research questions they felt were important. These research questions were selected to use open-ended responses to provide us with detailed insights from a respondent. The survey comprised of 17 multiple choice and 5 open-ended questions (Q8,9,10,15,17) and two open-ended responses in response to why someone choose an answer for Q4 and,5) (Supplementary Material 1). Ethics approval was by the Hunter New England Human Research Ethics Committee (ref no: 16/09/21/5.06). Consent was implied if participants returned their paper-based survey or completed the online survey.

### Participants

Participants were adults with stroke, TIA, or were carers for someone with stroke. Respondents reported whether they were a member of the Register or not. The survey was formatted so members of the Register had to complete questions specifically about the Register, then additional questions about reasons for participating in research. Non-members had only completed the additional questions about reasons for participating in research.

### Target sample size

A convenience sample of Register members, non-members who had experienced a stroke or TIA, or carers for an individual with stroke or TIA were invited to this study. Our inclusion criteria required participants to be over 18 years old, individuals with lived experience of stroke or TIA, were carers for someone with stroke. Register members were located within the Hunter Region, or the Hunter, a region in the state of New South Wales (NSW) Australia. Given the chosen dissemination strategy including social media platforms, responses were expected to be collected from across Australia and internationally. We had incorporated participants from outside of the Register to further understand what the population thought were important research themes and determine the barriers and drivers for individuals with stroke and their carers for participating in research (or not). As the first question requested for participants to identify as a member of the Register, we were able to identify responses from Register members. Based on central limit theorem, to be 95% confident that the answers from received surveys held a true representation of Register members, a minimum sample size of 81 members was required to achieve an accuracy within a 10% margin of error. With feedback from these 81 participants, we can be 95% confident that if 50% of the survey respondents answer a question in the same way, then 40–60% of all 513 Register members would answer similarly if they were to take the survey. Using a 5% margin of error, we would require a sample size of 220 Register members. With feedback from 220 Register members, we can be 95% confident that if 50% of the survey respondents answer a question in the same way, then 45–55% of all 513 Register members would answer similarly if they were to take the survey. Therefore, this study hoped to receive between 81 and 220 surveys from Register members to have a good representation of the Register. Additionally, feedback provided by non-members and carers was evaluated as data from a generalised group.

### Data analysis

Responses to multiple choice questions were reported as counts and percentages. A qualitative approach was taken for open-ended questions for the reporting to remain closer to the dataset while providing a comprehensive summary of the gathered information. Through a qualitative approach our researchers with lived experience of stroke could assist in the analyse of the data. Open-ended responses were therefore collated into Microsoft Excel to be thematically analysed using Braun and Clarke’s 6-stage process [9]. The authors familiarised themselves with the data (Step 1). Initial coding was completed by one researcher (JB), before being reviewed by two other researchers (CE and RP), one of whom also has lived experience of stroke (Step 2). Following this, the authors identified (Step 3), reviewed (Step 4), and defined (step 5) the themes. At each stage of the process, differences or conflicts in coding and themes was resolved by discussion amongst study authors (JB, CE, and RP).

## Results

A total of 445 people responded to the survey. Of these people, 406 completed the online survey and 39 returned a paper-based survey. Not all questions were answered by all respondents. Table 1 reports the total number of respondents for each question and the frequency of responses. For some questions, people could select as many answers as applied to them, therefore the percentages reported can add to > 100%.

**Table 1:** Responses to multiple choice questions

Question	Number of respondents	n (%)
What do stroke survivors value about being a member of the Register?		
Are you a member of the Stroke research Register	406	
Yes		154 (38)
No		168 (41)
I care for someone who had a stroke or TIA		55 (14)
Other		29 (7)
Why did you join the research register	138	
I want to test new treatments that might help me		74 (54)
I want to be informed about stroke research		100 (72)
Family member recommended it		6 (4)
Doctor/researcher/health professional recommended it		23 (17)
Not sure		3 (2)
Other		14 (10)
What do you like about being a member of the Stroke Research Register?	137	
Being asked to take part in research		92 (67)
Keeping up to date with research		104 (76)
Feeling part of a community of people with stroke or TIA		72 (53)
Hearing about community talks or events relevant to me		62 (45)
Other		2 (1)
How would you rate your experience of being involved in the Stroke Research Register?	114	
Excellent		61 (54)
OK		38 (33)
Not sure		12 (11)
Bad		1 (1)
I am not a member		1 (1)
What are stroke survivors’ top reasons for taking part (or not taking part) in research studies?		
Have you been invited to take part in any research studies about stroke? (number yes, %)	377	199 (53)
If yes, how many have you been invited to?		
not sure		44 (12)
0		119 (32)
1 to 3		149 (40)
4 to 5		13 (3)
> 5		12 (3)
How many studies have you participated in?		
0		184 (49)
1 to 3		136 (36)
4 to 5		9 (2)
> 5		9 (2)
Which things would help when deciding to take part in a study?	294	
The study might help me		197 (67)
The study might help others in the future		256 (87)
A person with stroke or TIA tells me what it was like in the study		44 (15)
Help with transport		51 (17)
Simple info about the study (plain language summary)		113 (38)
Detailed scientific info about the study		75 (26)
If you have decided not to take part in a study, why not?	214	
I was too busy		40 (19)
The study was asking too much of me		21 (10)
The information was too complicated		14 (7)
Transport was too difficult		43 (20)
I wanted to, but a researcher told me I wasn’t eligible		40 (19)
The study wasn’t relevant to me		46 (21)
Other		79 (28)
What are stroke survivors’ preferred communication methods with researchers?		
How did you find out about the study/studies?	107	
Letter or email from the Stroke Register team		44 (41)
Phone call from the Stroke Register team		18 (17)
Doctor / health professional		17 (16)
Radio / newspaper		6 (6)
Family / friend told me		14 (13)
Social media		29 (27)
Not sure		2 (2)
Other		12 (11)
How would you like to be invited to research studies?	303	
Letter or email from the Stroke Register team		272 (90)
Phone call from the Stroke Register team		89 (29)
Doctor / health professional		53 (17)
Radio / newspaper		12 (4)
Family / friend told me		47 (16)
Social media		76 (25)
Not sure		8 (3)
Other		7 (2)
How do you, or would you like to get stroke research information from our researchers?	297	
Regular newsletters (email or post)		274 (92)
Public talks / lectures about research		88 (30)
Community events with informal time to talk to a researcher		104 (35)
Other		14 (5)
Would you like to be more involved in:	286	
Deciding which issues are the most important to research (number yes, %)		208 (73)
Working with researchers to design better studies (number yes, %)		187 (65)

A total of 154 (38%) respondents reported that they were a member of the Stroke Research Register, and 168 (41%) reported they were not a member. The remainder reported being a carer of someone with stroke or TIA (*n* = 55, 12%) or answered ‘other’ (*n* = 29, 6%).

### Quantitative results

#### What stroke survivors’ value about being a member of the stroke research register

Most respondents reported wanting to be informed about stroke research (100/138, 72%) as being the main reason for joining the Register. Just over half (74/138, 54%) identified the opportunity to test new interventions as a reason they valued being a Register member. Only a small proportion of respondents joined on the advice of a doctor or heath professional (23/138, 17%). The most common perceived value of being Register member was keeping up to date with research (104/137, 76%), followed by being able to participate in research (92/137, 67%). Over half of respondents valued feeling part of a community of people with stroke or TIA (72/137, 53%). Most respondents rated their experience of the Register as excellent (61/114, 54%), or as good (38/114, 33%) and 128/134 (96%) of respondents said they would recommend others to join.

#### Stroke survivors’ top reasons for taking part (or not taking part) in research studies

199 of 377 (53%) respondents reported having been invited to participate in research studies. 256 of 294 responses (87%) indicated that their reason for doing so was that the study may help others in the future. Slightly fewer respondents (*n* = 197/294, 67%) chose to participate because they thought the study might help them. Reasons for *not* taking part in research studies were more varied and included the study not being relevant to them (*n* = 46/214 responses, 21%), difficulties with transport (*n* = 43/214 responses, 20%) or being too busy (*n* = 40/214 responses, 19%).

#### Stroke survivor and care preferred communication methods with researchers

Receiving a personalized direct letter or email from the Stroke Research Register team was the most common way respondents were invited to participate in studies (*n* = 44/107 responses, 41%), and their preferred communication method (*n* = 272/303 responses, 90%). Around a quarter of respondents (*n* = 29/107 responses, 27%) found out about studies via social media, and 76 people (of 303 responses, 25%) reported this as their preferred communication method. Almost all respondents wanted to receive regular newsletters (*n* = 274/297 responses, 92%), and one third also wanted to hear public talks (*n* = 88/297 responses, 30%) or community events (*n* = 104/297 responses, 35%).

##### Stroke survivors’ top research priorities

There were 113 free-text responses to the question *“Which are the most important problems related to stroke or TIA that we should be researching?”* These responses and themes are summarised in supplementary table (Supplementary Material 2). Most respondents wanted to be more involved in deciding which issues are important to research (*n* = 208/286 responses, 73%) and in working with researchers to design better studies (*n* = 187/286 responses, 65%). The most common research priorities were ‘Improving the rehabilitation experience’ (*n* = 86, 76%) and ‘Specific stroke impairments’ (*n* = 77, 68%). Additional research priorities included education (*n* = 32, 28%), movement recovery (*n* = 28, 25%), aphasia and communication, (*n* = 20, 18%) fatigue (n = 20, 18%), and mental health (n = 20, 18%).

##### Qualitative analysis of free-text responses

Several questions provided consumers the opportunity to provide a written response. These free-text responses were thematically analysed using and interpretive inductive process to identify key themes. The result of this process identified the following key themes: (i) *helping others*, (ii) *communication*, (iii) *barriers to participation in research* and (iv) *my research priorities*. The full coded free text responses and supporting quotes are presented in the supplementary material (Supplementary Material 2).

##### Helping others

Helping others was a key theme that emerged from participant responses. Register members regularly expressed a desire to help others with statements such as *“[I] also want to assist in the development of recovery techniques to help future generations”.* This was also seen as a way of giving back to the community of stroke survivors and health professionals working together to improve the lives of stroke survivors. “[*I want to] “contribute input that helped [them],[and] to help others.”* Concurrently, this was also seen as a way of finding their community because there was *“no where really to go except [the] GP after stroke and it feels reassuring to be part of a professional organization”*. However, respondents emphasised that it was an individual choice and ‘not for everyone’:

*“Personally, I would recommend other Stroke survivors but it is up to the individual.” “Every stroke and survivor is different.”.*

##### Communication

Poor communication was seen as a major area of concern that affected the experience of being a Register member. This was despite good experience with programs of research offered on the register.*“the programme was excellent, staff wonderful but the promised outcomes were never provided after the 6 or so months of excellent activity and assistance.”*. Stroke survivors also identified the importance that communication was a conversation and to not just one sided. *“I do want to participate but every time I ring the required number no one answers. It seems to me that everyone is busy including myself.”* However, when communication was effective, many stroke survivors reported that it helped them *“to be better informed about my stroke.*”

##### Barriers to participation in research

Stroke survivors identified several barriers for *not* taking part in research. This included issues with communication about the research with one participant describing how *“the whole study was a little bit too complex* Other stroke survivors reported on the energy demands of participation and that it could be *“too draining at the time for [them].” Geographical location of the research and* travel time was also a reported concern, *“I live in [a regional town] so it means return train travel & 2 night motel stay”* and how an increased flexibility in how trials are offered could improve participation “[It *would be good if my G.P. could do tests here and forward them on.”*. Additionally, previous negative experience could also be barrier to research participation. *“As a stroke survivor, participating in studies has generally lead to further feelings of isolation*. *.. it often feels more like a process of extracting information. .. more than a meeting in the middle.” “.*

##### My research priorities

There were many areas of research interest for stroke survivors as reported in the quantitative results. Many of the concepts revolved around research that could help the stroke survivor on an individual level such as education – ‘.

*“Whatever would have caused my stroke is still something I would like to know”, personalized care – “more work on individualization of stroke rehabilitation”*, and regaining movement *“Regaining movement of affected sides after rehab has finished.”.* However, other priorities were identified due to their widespread impact on stroke survivors including fatigue “*… one of the most important problems facing nearly all stroke survivors is fatigue”* and mental health *“psychology (clinical not neuropsych) interventions to help people recover from the trauma of stroke”.* Carers’ also identified key research priorities to support them and their role in caring for stroke survivors, “[There is a need to] m*aintain self-identity after stroke and assisting family cohesiveness in [a] time of immense stress.”*

## Discussion

We found that people with stroke valued the Stroke Research Register (Hunter) for its role in providing information about research, and for providing opportunities to participate. Furthermore, stroke survivors reported that they want to be involved in deciding important research priorities. The main motivations reported by stroke survivors and carers for participation in studies was because the study may help them or others in the future. Reported barriers to research participation included the study not being perceived as relevant to them, difficulties with transport to appointments, and a lack of time. While a quarter of people found out about research studies via social media, receiving a personal letter, email or phone call from researchers was the clearly preferred method of communication. For staying up to date with research, communication via paper or electronic newsletters was preferred. The most important research problems for stroke survivors and their carers included improving the rehabilitation experience through education and improved access to services, addressing specific impairments particularly recovery of movement, fatigue and aphasia, addressing mental health issues and increased support for carers support.

In this study, consumers contributed to the design of the survey, the development of the participant information, the design of promotional materials and the dissemination strategy. This partnership may have contributed to the large response to the survey and ensuring that the results are likely a good representation of the views of all Register members. Additional promotion of the survey via social media contributed responses from 252 people who were not Register members. Consequently, the results of the survey may be generalisable for Australia due to only 38% of participants being from the Register and 41% being non- registered members including some international responses.

While the current study identified the most important research problems, we found a similar observations in previously published research priorities seeking the perspectives of stroke survivors [10–13]. Other groups have published research priority statements based on key opinion leaders [14] or consensus from within groups of researchers [15–17]. Research priorities for consumers rarely include medically or scientifically driven themes [11, 12]. Therefore, it is essential to include their voice to ensure the most relevant research is conducted. Of the 3 published reports that set research priorities in partnership with consumers, the most comprehensive (input from stroke survivors, carers and health care professionals) was conducted in the UK in 2011 using the James Lind Alliance methodology [18]. There are striking parallels between the top 10 list of priorities arising from this work and the most frequently mentioned themes from our survey (see Table 2 and Supplementary Table 3). This shows that despite of the gap of a decade with the current study, the research priorities of consumers remain unchanged. The mismatch between what gets researched and what the consumers want researched was emphasized in several studies [19, 20]. The lack of change is concerning and studies addressing these key research priorities should be actively encouraged. The two smaller studies of consumer research priority setting for stroke [11] or TIA [12] included similar themes. This increases the confidence with which the research problems found in our study can be accepted despite limitations in the methodology used in this study. When asked, individuals with lived experience of any condition have a desire to partner with researchers in work that will affect them [21]. The respondents to our survey indicated a desire to be engaged in research priority setting and study design. The importance of genuine consumer partnership in research is now well recognized internationally [22, 23], and in many cases is an essential requirement for grant applications. Further, the current study supports the findings of the study of Healy et al. (2018) in identifying trial recruitment uncertainties [24]. Several priorities from this study are directly supported in the current study including consumer involvement in planning a randomised trial to improve recruitment, the best approaches for designing and disseminating information to consumers invited to participate in randomised trials and the barriers to participation in randomised trials.

**Table 2:** Comparison of our priority research themes with previous published work (common themes bolded)

Most frequently reported the research problems from our survey	Top 10 priorities for stroke, UK (Pollock et al., 2014)
**1. Education** for us and you	1. What are the best ways to **improve cognition** after stroke?
**2. Movement recovery (including walking, balance, arm function)**	2. What are the best ways to help people come to terms with the **long-term consequences** of stroke?
3. Improving rehabilitation services	3. What are the best ways to help people recover from **aphasia?**
**4. Aphasia/communication**	4. What the best treatments for **arm recovery and function?**
**5. Fatigue**	1. What are the best ways to treat visual problems after stroke?
6. Mental health / depression	6. What are the best ways to manage or prevent **fatigue?**
**7. Prevention of recurrent stroke**	7. What are the best treatments to improve **balance, gait and mobility**?
**8. Support for families and carers**	8. How can stroke survivors and **families be helped** to cope with speech problems?
**9. Living well long-term** after stroke	9. What are the best ways to **improve confidence** after stroke?
**10. Cognition**	10. Are exercise and fitness programs beneficial at improving function and quality of life and **avoiding subsequent stroke?**

Being involved in research gives participants a platform to voice their opinions on further research they would like to see in the future. This study has found a large proportion of individuals are interested in taking an active role in research. Involving participants in the development of future research may result in greater satisfaction with the research trial resulting in ongoing participation in future trials. Also, it empowers participants to take an active role in understanding their condition and feeling they had contributed to a greater cause. Being involved in research trials also may give people the feeling they were able to help themselves, other people with their condition, or assist stroke research to provide a better life for themselves and others. Researchers are encouraged to ask stroke survivors what they would like to see different in future research trials to design research that clinically addresses real problems.

The results from this survey can serve as a starting point for researchers to partner with consumer groups in developing relevant research studies. Building genuine consumer partnerships to design interventions and trials is challenging, and guidance for researchers to undertake it is limited. A handbook with case studies on how researchers and consumers have successfully used Integrated Knowledge Transfer methodology to achieve this has recently been published and includes examples of work with stroke survivors as well as other consumer groups [25].

### Strengths and limitations

Our study had both strengths and limitations. While we primarily targeted members of the Stroke Research Register in the Hunter Region, NSW, Australia, and did not collect demographic data of respondents, social media analytics showed that the survey was shared internationally. A small number of responses were from people living in the UK and the USA. The research priority data from this study is based on only one round of a survey with no input from health professionals or researchers. A research priority setting methodology should have been used to identify research priorities and to rank the research priorities. However, it does include responses from stroke survivors and carers. Our process of analyzing free text responses included coding by a researcher with lived experience of stroke which strengthens the relevance of these findings to consumers. Participant demographics would have added more understanding about the experiences and barriers to recruitment by sex, ethnicity, age, location and rurality. The responders may include motivated responders from the stroke register and those who are willing to be involved in research. Further, the lack of information on representative sample, survey fatigue and benefits and challenges of analysis of open-ended comments are acknowledged. There is a lack of detail on how representative the respondents are to the wider population, due to 41% of responses coming from non-members of the Register.

## Conclusion

In this survey of over 400 stroke survivors and carers, people with stroke reported a strong desire to be involved in research, not only as participants but as partners in research. These results suggest that many of the issues with trial recruitment that plague researchers could be ameliorated by engagement with consumers to ensure the research is of relevance to them and employing novel methods of trial delivery such as use of telehealth to reduce barriers around transport and time. The outputs of the survey have used in several ways to increase consumer engagement in the Register and two-way communication between members and research teams, link Register members with research teams as research partners, and supported trials focused on improving access by using telehealth approaches. Further research is recommended to find out how the current findings will be relevant to stroke survivors and how the research suggestions can overcome barriers in trial delivery. A qualitative study such as focus groups or interviews, or a research priority setting study relevant to Australian stroke survivors is recommended for more in-depth data about consumers perspectives. Importantly, there is an urgent need to address these consumers identified research priorities that were identified over a decade ago and yet remain unaddressed. Research collaboration and cooperation should be encouraged across networks to address the remaining gaps and improve the quality of life of stroke survivors.

## Supplementary Information

**Additional file 1.** Survey questionnaire.

**Additional file 2.** Key themes of free text responses.

**Additional file 3.** Research priorities.

## Data Availability

Data may be accessed upon reasonable request by contacting the corresponding author.
