# Supplementary material for: What do stroke survivors’ value about participating in research and what are the most important research problems related to stroke or transient ischemic attack (TIA)? A survey
Source: BMC Med Res Methodol. 2021 Oct 10;21:209. doi: 10.1186/s12874-021-01390-y (PMC8502417; doi:10.1186/s12874-021-01390-y)
Supplement: Supplementary file 1 — Additional file 1. Survey questionnaire. [file 12874_2021_1390_MOESM1_ESM.pdf]

Name

Address 1

Address 2 NSW XXXX

Survey. What do you think about the Stroke Research Register (Hunter)?

You can do the survey if:

1. You have had a stroke
2. You have had a TIA
3. You care for someone who has had a stroke or TIA

It will take about **ten (10) min**

There are **seventeen (17) questions**

We want to find out what you think about

- The Stroke Research Register, and
- How we as researchers can work better with you to improve the lives of people with stroke and TIA.

Thank you - prize draw:

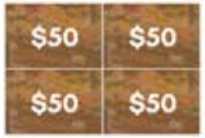

Four (4) people who do the survey will **win a $50** supermarket voucher.

The Stroke Research Register connects people who have had a stroke or transient ischaemic attack (TIA) with researchers in the Hunter region. Register members get invitations to take part in research studies over time. This helps researchers from the University of Newcastle, Hunter Medical Research Institute (HMRI) and Hunter New England Local Health District (HNEHealth) test new treatments and rehabilitation approaches for stroke and TIA. There are now 500 people registered.

First - please read the info booklet

It says who we are and explains how we will use your answers.

Do the survey – use this form, or

Do the survey online http://bit.ly/10minstrokesurvey

Thank you, from the research team for this survey (University of Newcastle).

Survey form.

Want help?
4042 0093 or strokeregister@hmri.org.au

☐ I have had a stroke or TIA, or care for someone who has

☐ I am 18 years or older

☐ I have read and understood the enclosed detailed information about how my answers will be used

1. Are you a **member** of the **Stroke Research Register?**

- ☐ Yes ✓
- ☐ No ✗ → **go to Question 6**
- ☐ Not sure **?** → **go to Question 6**
- ☐ I **care for someone** who had a stroke or TIA → **go to Question 6**
- ☐ Other ✎ ______________ → **go to Question 13**

If you **are a member** of the Stroke Research Register, answer Q 2-5.

2. **Why did you join?** Mark as many as apply to you

- ☐ I want to **test new treatments that might help me**
- ☐ I want to be **informed about stroke research**
- ☐ **Family** member recommended it
- ☐ **Doctor** / researcher / health professional recommended
- ☐ **Not sure ?**
- ☐ Other ✎ ______________

3. **What do you like** about being a member of the Stroke Research Register?

- ☐ Being asked to **take part** in research
- ☐ Keeping **up to date** with research
- ☐ Feeling **part of a community** of people with stroke or TIA
- ☐ Hearing about community **talks or events** relevant to me
- ☐ Other ✎ ______________

4. How would you **rate your experience** of being involved in the Stroke Research Register?

- ☐ **Excellent** ☺☺☺
- ☐ **OK** ☺
- ☐ **Not sure ?** 😐
- ☐ **Bad** ☹
- ☐ I'm **not a member**

Why did you chose this rating?

____

5. Would you **recommend other people with stroke or TIA** join the **Stroke Research Register?**

- ☐ Yes ✓
- ☐ No ✗
- ☐ Not sure **?**

Why?

____

6. **Have you been invited** to take part in any **research studies** about stroke?

- ☐ Yes ✓
- ☐ No ✗
- ☐ Not sure **?**

How many have you been **invited to**?

Not sure | 0 | 1 - 3 | 3 – 5 | >5

How many have you been **participated in**?

0 | 1 - 3 | 3 – 5 | >5

7. If you have **decided not to take part** in a study, **why not?**
Select all that apply to you

- ☐ I was **too busy**
- ☐ The study was **asking too much of me**
- ☐ The info was **too complicated**
- ☐ **Transport** was too difficult
- ☐ I wanted to, but a **researcher told me I wasn't eligible**
- ☐ The study **wasn't relevant** to me
- ☐ Other ✎ ______________________

If you have participated in any research studies:

☐ ✖ I have never taken part in a study ➔ **go to Q12**

8. Why did you take part?

____

9. What did you like about the study or studies? ☺

____

10. Was there **anything you didn't like?** ☹

____

11. How did you find out about the study / studies?
Mark all that apply to you

- ☐ **Letter or email** from Stroke Register team
- ☐ **Phone** call from Stroke Register team
- ☐ **Doctor** / health professional
- ☐ **Radio / newspaper**
- ☐ **Family / friend** told me
- ☐ **Social Media**
- ☐ **Twitter**
- ☐ **Instagram**
- ☐ **Not sure**
- ☐ Other ✎ ______________________

12. Which things would help when deciding to **take part in a study**?
Mark all that apply to you

- ☐ The study might **help me**
- ☐ The study might **help others** in the future
- ☐ A **person with stroke or TIA tells me what it was like** in the study
- ☐ Help with **transport**
- ☐ **Simple info** about the study (plain language summary)
- ☐ **Detailed scientific info** about the study

13. How would you like to be invited to research studies?
Mark as many as you like

- ☐ **Letter or email** from Stroke Register team
- ☐ **Phone** call from Stroke Register team
- ☐ **Doctor** / health professional
- ☐ **Radio / newspaper**
- ☐ **Stroke survivors**
- ☐ **Social media**
- ☐ **Not sure**
- ☐ Other ________________

14. How do you, or would you like to get stroke research information from our researchers?

Select all that apply to you
- ☐ Regular newsletters (email or post)
- ☐ Public talks / lectures about research
- ☐ Community events – with informal time to talk to a researcher
- ☐ Other ________________

15. Which are the **most important problems related to stroke or TIA** that **we should be researching?**

These might be:

- Problems you have since your stroke or TIA
- Problems other people have since their stroke or TIA
- Problems faced by carers of people with stroke or TIA
- Something else

16. Would you like to be more involved in
You can **choose Yes or No**
- Deciding which issues are the most important to research ☐ Yes ☐ No
- Working with researcher to design better studies ☐ Yes ☐ No

17. Is there anything else you'd like to tell us?

END.

Don't write your name on it.

Send your form in the reply paid envelope.

To enter the prize draw **4042 0093 or**

e: strokeregister@hmri.org.au

Thank you for participating. A summary of the survey results will be published in a future Stroke Research Register newsletter.
