# Supplementary material for: What do stroke survivors’ value about participating in research and what are the most important research problems related to stroke or transient ischemic attack (TIA)? A survey
Source: BMC Med Res Methodol. 2021 Oct 10;21:209. doi: 10.1186/s12874-021-01390-y (PMC8502417; doi:10.1186/s12874-021-01390-y)
Supplement: Supplementary file 2 — Additional file 2. Key themes of free text responses. [file 12874_2021_1390_MOESM2_ESM.docx]

Supplementary Table 2. Key themes of free text responses

| Research question | Survey question | Themes | Supporting quotes |
| --- | --- | --- | --- |
| What do stroke survivors’ value about being a member of the Stroke Research Register? | Why did you join the Register? | To help others now and in the future | *“I also want to assist in the development of recovery techniques to help future generations.”*  “*I'd like to contribute input that helped me, to help others.”*  *“If my experience and information can help then that's excellent. I survived from people helping me. Just want to give something back.”* |
|  |  | Help myself and learn something | *“Desire to improve my condition.”*  *“I am keen to know how stroke survivors are continually supported and therefore interested to know how people are given opportunities to be involved in their own recovery.”*  *“Mainly to find out what could be done to improve my recovery.”* |
|  |  | Paying it back | *“Payback for the excellent care I received at xxxx.”*  *“I'd like to contribute input that helped me, to help others.”* |
|  |  | Set research priorities | *“I want to be involved in setting research priorities.”* |
|  | How would you rate your experience of being involved in the Stroke Research Register? | Feeling supported and part of a community | *“No where really to go except GP after stroke and it feels reassuring to be part of a professional organization.”*  *“The people I have [dealt] with have been great & understand the position that I am in.”* |
|  |  | Paying it back | *“If my experience and information can help then that's excellent. I survived from people helping me. Just want to give something back.”* |
|  |  | To gain information | *“Mainly to find out what could be done to improve my recovery.”*  *“Because it's great learning more things about stroke/Aphasia.”* |
|  |  | Positive experience in research | *“The [deleted] study has helped me a lot”.*  *“I enjoyed meeting so many people who were passionate about their jobs, and I was impressed by the care they took to make everything easy for me.”*  *“Because I feel I am being listened to.”* |
|  |  | Lack of communication or involvement | *“Had difficulties with communication.”*  *“The programme was excellent, staff wonderful but the promised outcomes were never provided after the 6 or so months of excellent activity and assistance.”*  *“Because I haven't had much experience with it as yet.”* |
|  | Would you recommend others to the research register? Why? | Keep informed | *“It raises your awareness and causes you to consider what can be done post TIA in my case.”*  *“Worth joining for possible help knowledge of stroke recovery news and trials importan.t”*  *“I think it is a good way to be informed without any pressure.”* |
|  |  | Meet others in the stroke community | *“To be involved, maybe gain some beneficial help, meet others in the stroke community.”*  *“It is good to be involved to meet other survivors and to help.”* |
|  |  | To help myself and others | *“Extra advice, feeling that I am helping others down the track”*  *“Because it may be beneficial to our recovery”*  *“Motivation. A sense of hope”* |
|  |  | To help research and develop new treatments | *“Anything to further research is important”.*  *“The more we understand stroke and stroke recovery the better the treatment in the future.”*  *“It all helps the researchers, the patients and future generations.”* |
|  |  | It’s not for everyone | *“Up to them after discussion.”*  *“Personally I would recommend other Stroke survivors but it it is up to the individual.”*  *“Every stroke and survivor is different.”* |
| What are stroke survivors’ top reasons for taking part (or not taking part) in research studies? | If you have participated in any research - why did you take part? | I thought it might help | *“To try and improve my abilities and learn about preventing further strokes.”*  *“Thought it might help my recovery.”*  *“A bit of self-interest, but really wanting to find what could be done to help me in the future.”*  *“To be better informed about my stroke.”* |
|  |  | To help others | *“To help myself and others.”*  *“I have been lucky after my TIA. I thought the study may help others.”*  *“To improve your own recovery and to help future stroke survivors.”* |
|  |  | To help research | *“Very interested in research based studies.”*  *“To help researchers/others and myself.”*  *“Maybe I could help with your research.”*  *“Interest in science.”* |
|  | If you have participated in any research studies, what did you like? | It was easy and enjoyable | *“Interview and participation activities were relevant and easy to understand.”*  *“Some of it was like playing games.”*  *“Always liked being part of the studies as I get quite a lot myself in return by belonging to a group.”* |
|  |  | Friendly staff/researchers | *“People were supportive and friendly.”*  *“Enjoyed company of researchers. Got me out of the house. I contributed to studies.”*  *“Treated kindly + consulted about what would happen. Liked follow up information.”* |
|  |  | Feedback and information | *“Monitoring ie blood pressure, monitor on body. This made me aware of my activities. Having to answer quizzes which revealed to me personally where my mental acumen (ie sharpness of the mind) was at.”*  *“Exercises ; feedback was thorough + performed + useful + practical for ongoing knowledge.”*  *“I've learned a lot of things I didn't know.”* |
|  |  | Helping "pay it forward" | *“Helping those that helped me.”*  *“That my episode could help others.”* |
|  | If you decided not to take part in a research project, why not? | Poor communication | *“I do want to participate but every time I ring the required number no one answers. It seems to me that everyone is busy including myself. Work is busy.”*  *“I didn't here (sic) back from you.”*  *“Never asked but I would like to.”*  *“The whole study was a little bit too complex for me. (Would like to know more before I jump in).”* |
|  |  | My body just wouldn’t cope | *“Other health problems have prohibited my participation, due to mobility issues.”*  *“Too draining at the time for me.”*  *“Complications from Stroke prevented me attending at 'set' times.”*  *“The study required me to be sedentary for too long and my body just wouldn't cope.”* |
|  |  | Travel and time | *“I live in xxx so it means return train travel & 2 night motel stay.”*  *“Moved to xxx. Too far away.”*  *“Was hard to take time off from work when I needed to be at the institute.”* |
|  |  | Negative past experience | *“As a stroke survivor, participating in studies has generally lead (sic) to further feelings of isolation. Language used is too often reductive or ostracising and it often feels more like a process of extracting information (underpinned by a top-down paradigm) more than a meeting in the middle, leveraging lived experiences on for all parties, and building trusting & empowering pathways forwards, together.”* |
|  |  | Poor communication from Register | *I do want to participate but every time I ring the required number no one answers. It seems to me that everyone is busy including myself. Work is busy.*  *I didn't here (sic) back from you*  *Never asked but I would like to*  *The whole study was a little bit too complex for me. (Would like to know more before I jump in)* |
|  |  | Don't feel able to | *Other health problems have prohibited my participation, due to mobility issues.*  *Too draining at the time for me*  *Complications from Stroke prevented me attending at 'set' times.*  *The study required me to be sedentary for too long and my body just wouldn't cope.* |
|  |  | Travel and time | *I live in xxx so it means return train travel & 2 night motel stay.*  *Moved to xxx. Too far away.*  *Was hard to take time off from work when I needed to be at the institute* |
|  |  | Negative past experience | *As a stroke survivor, participating in studies has generally led to further feelings of isolation. Language used is too often reductive or ostracising and it often feels more like a process of extracting information (underpinned by a top-down paradigm) more than a meeting in the middle, leveraging lived experiences on for all parties, and building trusting & empowering pathways forwards, together.* |
|  | Was there anything you didn't like (about being involved in research)? | Transport and time commitment | *“Hour’s drive but I got used to it.”*  *“Schedule may be difficult.”* |
|  |  | Communication issues | *“Slight lack of communication about wearing the monitor.”*  *“Have not received any feedback.”*  *“There are no process to get positive result to health professional so they can apply it to their clients.”* |
|  |  | Testing or activities involved | “*The shapes-and-colours one that had that terrible, condescending American voice.”* |
| What are stroke survivors’ preferred communication methods with researchers? | How would you like to get stroke research information and updates from our researchers? | Through community groups and events | *“Through the Aphasia group I am part of.”*  *“Maybe an open day for the teams & people on the register would be a good thing.”* |
|  |  | Non face-to-face methods | *“Hard to get to events!! Can't drive and limited mobility present as problems to venues.”*  *“I do enjoy following doctors, providers & specialists who focus on stroke recovery online (on social media) I like seeing their updates on Twitter.”* |
