# Supplementary material for: What do stroke survivors’ value about participating in research and what are the most important research problems related to stroke or transient ischemic attack (TIA)? A survey
Source: BMC Med Res Methodol. 2021 Oct 10;21:209. doi: 10.1186/s12874-021-01390-y (PMC8502417; doi:10.1186/s12874-021-01390-y)
Supplement: Supplementary file 3 — Additional file 3. Research priorities. [file 12874_2021_1390_MOESM3_ESM.docx]

Supplementary Table 3. Research priorities

| Theme | n (%) | Sub-theme | n (%) | Quotes |
| --- | --- | --- | --- | --- |
| Improving the rehabilitation experience | 86 (76) | Education for us and you | 32 (28) | *“Others recognising the signs. When I had a tia people thought I was intoxicated”*  *“information about after stroke problems and possible ways of dealing with problems”*  *“... but I think it could also be worth investing research time/money into identifying/celebrating solutions & successes which survivors' experience - and working from that point first.”* |
|  |  | Improving rehabilitation services | 22 (19) | *“the transition from facility to home, preparedness of family, and the environment”*  *“more work on individualisation of stroke rehabilitation”* |
|  |  | Prevention of recurrent stroke | 15 (13) | *“Exercise and its effect on recovery and prevention of future strokes”*  *“Follow up when you have had a TIA/stroke and any information that would help in preventing another episode”* |
|  |  | Living well long-term after stroke | 14 (12) | *“Energy levels long term, Long term issues that may occur”*  *“Post stroke recovery long term”* |
|  |  | Accessibility of care | 3 (3) | *“Would like to see programs developed that address some of these ideas via telehealth so stroke patients can access”*  *“The online exercise research looks promising for people that don't have access to places like xxx Services which has been great for me.”* |
| Specific stroke impairments | 77 (68) | Movement recovery (walking, balance and arm function) | 28 (25) | *“Moving again after stroke”*  *“Regaining movement of affected sides after rehab has finished.”*  *“Getting the use of affected limbs as quickly as possible”* |
|  |  | Aphasia/communication | 20 (18) | *“Problems I have is frustration of not having able to do things like writing this. I have to get my mother to do it for me. I have to have all my paperwork checked by someone this is frustrating. Not understanding the questions and having to ask someone all the time.*  *“Problems understanding information (l like to know treatment / studies [about] living with Aphasia I have trouble processing the information)”*  *“Aphasia!!!”* |
|  |  | Fatigue | 20 (18) | *“I suffer fatigue and I'm a little over some doctors blaming any further health issues on it was probably from your stroke.”*  *“…one of the most important problems facing nearly all stroke survivors is fatigue”*  *“FATIGUE ISSUES!! Especially in those that don't have outward showing signs of stroke ie no paralysis or aphasia”*  *“trial something else other than modafinal for post stroke fatigue”* |
|  |  | Pain and spasticity | 8 (7) | *“For me personally neurological pain and spasticity plus endless fatigue.”*  *“Post stroke Thalamic Pain [also known as Central Pain] and Spasticity.”* |
| Mental health and cognition | 39 (35) | Mental health / depression | 20 (18) | *Every day is an effort to accept who/where I am & when close supports try to encourage me to "go back" I feel as though there's something wrong with where I am.*  *“Not enough mental health available to access”*  *“Most effective ways of linking survivors to other survivors to reduce loneliness/isolation. Research into the experience of stroke as 'trauma'”* |
|  |  | Cognition | 10 (9) | *cognitive rehabilitation delivered via telehealth*  *“Managing evolving effects post-stroke - e.g. cognitive decline in years following the event.”*  *“Best practice/support in navigating return to work whilst managing cognitive, communication and fatigue issues”* |
|  |  | Memory | 9 (8) | *“memory loss”*  *“Ways of helping stroke victims deal with and improve short term memory and facial recognition.”* |
| Improving care early after stroke | 20 (18) | Improving early stroke care | 11 (10) | *“Methods of detecting a brain injury in a hospital.”*  *“Headaches are not taken seriously then people have strokes or die by Drs & emergency departments”*  *“Education for everyone include hospital staff as my first stroke took xxx hospital three days to identify so frontline needs to improve”* |
|  |  | Medical advancements | 9 (8) | *“effect of oxygen therapy on brain to assist the healing process.”*  *“How long after the stroke it is still appropriate to give the thrombolytic drug”*  *“Stem cells”* |
| Support for carers/family | 15 (13) |  | 15 (13) | *“Can't leave carers out when dealing with someone living with carer. Big impact on family + carer.”*  *“Family co-cohesiveness after stroke.”*  *“carers require techniques to handle conflicts/difficulties/general day to day tasks of stroke sufferers”* |
| Young stroke/return to work | 8 (7) |  | 8 (7) | *“How to explain to others (e.g. people at work) what has happened and what they may see as a result of me having a stroke”*  *“Going back to work is my current struggle. I can't do the physical work I was doing before & I can't manage the high stakes/multitasking demands of jobs I had previously.”*  *“Young stroke survivors and impact of stroke on relationships and returning to work.”* |
